# Goals in Nutrition Science 2015–2020

**DOI:** 10.3389/fnut.2015.00026

**Published:** 2015-09-08

**Authors:** David B. Allison, Josep Bassaganya-Riera, Barbara Burlingame, Andrew W. Brown, Johannes le Coutre, Suzanne L. Dickson, Willem van Eden, Johan Garssen, Raquel Hontecillas, Chor San H. Khoo, Dietrich Knorr, Martin Kussmann, Pierre J. Magistretti, Tapan Mehta, Adrian Meule, Michael Rychlik, Claus Vögele

**Affiliations:** ^1^Office of Energetics and Nutrition Obesity Research Center, School of Public Health, University of Alabama at Birmingham, Birmingham, AL, USA; ^2^Section on Statistical Genetics, University of Alabama at Birmingham, Birmingham, AL, USA; ^3^Department of Nutrition Sciences, University of Alabama at Birmingham, Birmingham, AL, USA; ^4^Department of Biostatistics, University of Alabama at Birmingham, Birmingham, AL, USA; ^5^Nutritional Immunology and Molecular Medicine Laboratory, Virginia Bioinformatics Institute, Virginia Tech, Blacksburg, VA, USA; ^6^Deakin University, Melbourne, VIC, Australia; ^7^American University of Rome, Rome, Italy; ^8^Nestlé Research Center, Lausanne, Switzerland; ^9^Organization for Interdisciplinary Research Projects, The University of Tokyo, Tokyo, Japan; ^10^École Polytechnique Fédérale de Lausanne, Lausanne, Switzerland; ^11^Institute of Neuroscience and Physiology, The Sahlgrenska Academy at the University of Gothenburg, Gothenburg, Sweden; ^12^Department of Infectious Diseases and Immunology, Faculty of Veterinary Medicine, Utrecht University, Utrecht, Netherlands; ^13^Faculty of Science, Utrecht Institute for Pharmaceutical Sciences, Utrecht University, Utrecht, Netherlands; ^14^North American Branch of International Life Sciences Institute, Washington, DC, USA; ^15^Technische Universität Berlin, Berlin, Germany; ^16^Nestlé Institute of Health Sciences SA, Lausanne, Switzerland; ^17^Division of Biological and Environmental Sciences and Engineering, King Abdullah University of Science and Technology, Thuwal, Saudi Arabia; ^18^Laboratory of Neuroenergetics and Cellular Dynamics, Brain Mind Institute, École Polytechnique Fédérale de Lausanne, Lausanne, Switzerland; ^19^Department of Health Services Administration, Nutrition Obesity Research Center, University of Alabama at Birmingham, Birmingham, AL, USA; ^20^Department of Psychology, University of Salzburg, Salzburg, Austria; ^21^Analytical Food Chemistry, Technische Universität München, Freising, Germany; ^22^Research Unit INSIDE, Institute for Health and Behaviour, University of Luxembourg, Luxembourg, Luxembourg

**Keywords:** nutrition, food, sustainable development, big data analysis, food safety, behavior, brain health, human microbiome

With the definition of goals in Nutrition Science, we are taking a brave step and a leap of faith with regard to predicting the scope and direction of nutrition science over the next 5 years. The content of this editorial has been discussed, refined, and evaluated with great care by the Frontiers in Nutrition editorial board. We feel the topics described represent the key opportunities, but also the biggest challenges in our field. We took a clean-slate, bottom-up approach to identify and address these topics and present them in eight categories. For each category, the authors listed take responsibility, and deliberately therefore this document is a collection of thoughts from active minds, rather than a complete integration or consensus.

At Frontiers in Nutrition, we are excited to develop and share a platform for this discussion. Healthy Nutrition for all – an ambition too important to be handled by detached interest groups.

Johannes le Coutre, Field Chief Editor, Frontiers in Nutrition

## Sustainable Development Goals for Food and Nutrition

(Barbara Burlingame, Chor San H. Khoo, and Dietrich Knorr)

To deliver successfully, nutrition research needs a bold dose of innovation. Moving forward from the Millennium Development Goals to the post-2015 sustainable development goals (SDG), global nutrition appears to require an improved model. Under current practices, feeding the exploding world population necessitates to close a gap of nearly 70% between the amount of food available today and the projected availability by 2050 (1). Today, globally, an estimated 805 million people are undernourished or food insecure (2), yet one out of four calories from food goes uneaten. Meanwhile, overweight and obesity affect approximately two billion people, including 42 million children under the age of 5. Human health notwithstanding environmental health is also at stake. Agriculture alone accounts for about 70% of our global water usage and 24% of our greenhouse gas emissions. As a result, our strategies to overcome issues of food sustainability, food waste, and food loss must be multifarious and include, at the very least: (i) Improving the global consumption of food. (ii) Increasing production efficiencies on existing agricultural land. (iii) Developing sustainable approaches that reduce the environmental impact of food production, and in particular greenhouse gas emissions. Certainly, the impact of agriculture on climate, ecosystems, and water will have to be reduced, while at the same time, we will need to ensure that it supports inclusive economic and social development (1).

Systems science, the interdisciplinary field that explores the nature of complex systems, is perhaps the best research model we have for addressing the urgent needs of a precariously unhealthy planet. For better or for worse, nutrition imparts a quintessential challenge, straddling many sectors and disciplines.

In the past, at times, the agenda for mainstream nutrition has been pushing sectoral lines of reasoning by implementing policies that leave long-standing problems unresolved, while disrupting other sectors in the process. Of course, nutrition is not alone in this, but the history of unintended consequence is long and discouraging (3, 4).

Agriculture and health have been the mainstay sectors at the United Nations level, in government ministries, and in academic departments. Increasingly, nutrition is being recognized as an important pillar for the environmental sector, with biodiversity for food and nutrition acknowledged by the Convention on Biological Diversity (5), and the Commission on Genetic Resources for Food and Agriculture accepting whole diets, food, and nutrients for human nutrition as ecosystem services (6).

For all their embracing of nutrition, these sectors often work at cross-purposes, providing many useful illustrations of policies and programs that undermine each other’s development efforts. We have policies and interventions in agriculture that contribute to diet-related chronic disease, environmental degradation, and food insecurity (4, 7); conversely, in the health sector we have policies and interventions that compromise agricultural development (8); and in the environmental sector that lead to micronutrient malnutrition (9). Agriculture in particular, while solving some of its own sector problems, has been associated with many of the environmental and human health crises we now face, which directly impact upon nutrition, including chemical contamination of food supplies, loss of agrobiodiversity, and severe environmental degradation (10).

In spite of the clear need to develop innovation for the future, “*systematic attempts to explore existing methods and to develop new technologies of more sustainable food production systems have so far been scarce*” (11). Although this quote is from over 30 years ago, it still quite accurately describes the current situation regarding activities related to sustainable diets and sustainable food systems. A sustainable development lens with a systems science approach offers not only a new analytical model for nutrition, but also an ethical and inclusive framework. Within this framework, nutrition encompasses more than its traditional domains and takes on issues of climate change (12), biodiversity and ecosystems (13), water use/waste (14), food losses and waste (15, 16), sustainable forests and seas (17), chemical contamination of food and water supplies (18), environmental regulatory issues and food law, risk and risk/benefit assessments (19), and monitoring adherence to and compliance with a range of relevant treaties and signed declarations/commitments (13).

With this mindset of sensitive, cross-sectoral resolve, tangible and specific solutions will envisage a holistic food chain integration taking into account a total life cycle assessment. Food and nutrition security must be an intrinsic component of any solution for food sustainability. Forthcoming strategies will also have to explore the potential and utilization of new raw materials.

Improvements of food safety, storage, packaging, and transportation – including the use of sensor technologies – can reduce food losses and waste. Innovation will have to equally encompass the re-evaluation of existing food processing, storage, and home preparation operations employing existing modern toolboxes. Moreover, low energy, waste-free or waste-reduced processing, and preparation operations need to be implemented to a larger extent, including alternative energy sources. In the same context, water decontamination, recycling, and preservation tools need to be applied.

Unintended consequences must be considered with any sustainability program and global solutions are not necessarily applicable in local contexts. For example, reducing livestock production and consumption in one setting may benefit both human and environmental health, while in another setting it may reduce further already marginal intakes of high-quality protein and micronutrients and marginalize grazing lands that are self-renewing, sustainable repositories of biodiversity. Finally, young engineers and scientists need to be encouraged, trained, and involved to tackle the challenges of the future.

We have a planet in crisis on so many fronts. Regardless of how the SDGs evolve, this multi-sectoral vision of nutrition research and action has the potential to make meaningful, and sustainable, contributions.

## Identifying and Mitigating Errors in Nutritional Science

(David B. Allison, Andrew W. Brown, and Tapan Mehta)

“Science,” as Adam Smith famously said, “is the great antidote to the poison of enthusiasm and superstition” (20). Complementarily, Stephen Hawking has called scientists, “the bearers of the torch of discovery in our quest for knowledge” (21). Thus, science can be seen as having two key complementary roles – dispelling false beliefs, and creating new knowledge. For science to fulfill this joint mission, its practice must be true to its principles and precepts, including objectivity, methodological rigor, transparency, and reproducibility. Yet, there are concerns that departures from these precepts are too common (22–28). Some have speculated that deviations from good scientific practices have increased in recent years due to a number of social, institutional, and economic factors in science (25, 29). Others have speculated that the problem may be especially severe in the related domains of nutrition research and obesity research, perhaps because of emotional, economic, and other factors involved in those topics or because the everyday familiarity with aspects of those topics is mistaken for expertise (23, 26–28). It is difficult to quantify whether the situation is better or worse today than in the past, or whether this is especially true in nutrition and obesity research compared to other fields. Nevertheless, it is clear that the problem exists.

Several initiatives are going to be important in the coming years to improve nutrition as a science. First is classifying errors that exist in the nutrition literature. Just as Mendeleev’s Periodic Table of the Elements led to increased understanding of chemistry and Linnaeus’ taxonomy of life led to a framework for the study of biology, if we can develop a “pathology” or classification of these errors, we may be better able to quantify the situation, identify patterns, develop an understanding of origins, and ultimately reduce the occurrence and severity of these errors. In our non-systematic study of these issues, we see a number of categories of common errors (Table 1). We refer to them as errors without making any inference that they are intentional or unintentional errors.

**Table 1:** **Common errors noted in the published literature^a^**.

Error	Example(s) of error
Errors involving or resulting from poor measurement	• Self-reported energy intake (33, 118, 119)^b^ (34)^c^ (32)^d^
	• Self-reported weights (120)^b^ (121, 122)^d^
Errors involving inappropriate choice of or incorrect study design	• Cluster randomized trials with no degrees of freedom (123)^c^
	• Lack of control for non-specific factors, i.e., failure to isolate the independent variable of interest (124)^c^
	• Non-random assignment in self-described RCTs (125)^b^
Errors involving replication	• Not validating prediction models in fresh samples (126)^d^
	• Gratuitous replication (35)^d^
Errors in statistical analyses	• Inappropriate baseline testing in parallel groups RCTs (127)^c^ (128)^d^
	• Failure to appropriately manage missing data (129)^c^ (130, 131)^d^
	• Not accounting for clustering in cluster randomized trials (132, 133)^b^ (134, 135)^c^ (136)^d^
Errors involving insufficient transparency in choices made about how to analyze and present the data	• Changing endpoints in a study (137)^b^ (138)^d^
	• Excessive or unacknowledged multiple testing [called p-hacking (139)^d^, investigator degrees of freedom (140)^d^, or p-value fiddling (141)^d^, among other names] (142)^c^ (143)^b^
Errors of misleadingly describing past literature	• Selectively citing only the part of a study that supports a hypothesis (35)^d^
	• Perpetuating citations from previous research without confirming the original source (144)^b^
Errors that distort the scientific record by publishing studies as a function of study outcomes	• Publication bias (145)^b^ (23, 146)^d^
Errors of interpretation or communication	• Inappropriate use of causal language (24, 35)^d^
	• Exaggerating or mis-describing results (35)^d^
	• Highlighting benefits of treatment when the effects were non-significant (i.e., spin) (147)^d^
	• Issuing misleading press-releases (148)^d^
Errors of logic and mathematics	• Unreasonable linear extrapolations (e.g., 3500 kcal rule) (149, 150)^b^

*^a^Errors, examples, and references were identified in a manner neither systematic nor comprehensive*.

*^b^Denotes references correcting or commenting on specific errors*.

*^c^Denotes references in which the error in question occurred*.

*^d^Denotes references that provide tutorials on avoiding or overviews of the errors*.

Second, there is a general movement in science for “Transparency and Openness Promotion,” formalized in “The TOP Guidelines” (30). The guidelines recognize eight standards: citation, data transparency, analytic methods (code) transparency, research materials transparency, design and analysis transparency, preregistration of studies, preregistration of analysis plans, and replication. These standards aim to improve the communication of science, allowing improved understanding and replicability of results. Because the TOP Guidelines are being adopted across fields of science, the field of nutrition will not have to act in isolation to improve its scientific practices. Instead, we can build on and work with the minds and resources coming from a spectrum of scientific inquiry. Indeed, *Frontiers in Nutrition* was one of the initial signatories.

Third, there is a need to develop sound methodology for evaluating nutrition and diet in free-living research participants. Issues are continually documented with self-report diet methodology (31–33), and yet dietary recommendations depend heavily on dietary recall data (34). Similarly, although existing nutrition-related health hypotheses can be investigated using randomized controlled trials (pragmatic or explanatory), the field often relies on ordinary association tests using observational data to quantify evidence (35, 36) that policy-makers may then use to create policies or guidelines. The needs here are twofold: to develop and implement study designs that lie in the causality spectrum between ordinary association tests and randomized controlled trials (37, 38) and to develop objective, reliable data on dietary patterns and nutrient status (31–33).

We believe that by recognizing and acknowledging these problems, we also recognize and acknowledge that our field can do better. This will pave the way toward constructive efforts to reduce such problems and to ultimately improve the scientific foundations of nutrition science.

## Building the Foundation: Procurement of Relevant Measures and Big Data Analysis

(Martin Kussmann, Josep Bassaganya-Riera, Raquel Hontecillas, Tapan Mehta, and Chor San H. Khoo)

Diet is considered a key environmental factor for maintaining health and preventing disease. As such, we need to better understand the interactions of nutrition and lifestyle with an individual’s genetic makeup in order to delay or prevent metabolic and cognitive decline. Nutrition science is therefore undergoing a paradigm shift to better leverage the potential of nutrigenomics, a discipline that is already transforming the field (39). To achieve this, the field will need to transform its current approach to research and implementation actions, and to take advantage of emerging advances in other disciplines – research designs, methods, new technologies, big data analysis, and bioinformation sharing.

The conceptual basis of gene – environmental interactions require not only research and technology, but also the cross-fertilization of disciplines: genomics will encompass other-omics, and nutrition research will need to take on a holistic or system biology approach rather than just nutrients, ingredients, or genes. Nutrition science now encompasses more than the classic reductionist and descriptive approaches to more quantitative and systems-level approaches (40). Translational research to maintain health and prevent or delay disease onset requires a *transdisciplinary* approach that embraces the complexity of human individuality in a rapidly changing environment. Nutrigenomics fuels this research by investigating how genomic and epigenomic individuality predisposes dietary, health, and disease responses. It also influences how an individual’s genome expresses itself at different omic levels (proteomics, metabolomics, lipidomics) in response to environmental factors, including nutrition. Molecular phenotyping of humans over time and across healthy and safe exposures and challenges have thus been proposed (41).

Both the ongoing prevalence of malnutrition and the increasing incidence of nutrition- and lifestyle-related chronic diseases require comprehensive characterization of the complex interactions between environment and genetic makeup. Systems thinking in human nutrition, environment, and health requires improvement and translational thinking in three areas:

(a) *In vitro* and *in vivomodels*: a systems approach to human health implies rethinking of *in vitro* and *in vivo* models with regard to their translatability into human phenotypes: natural human cell models and panels of rodent strains should complement cancer cell lines and single rodent strains.
(b) *Human intervention study designs:* classical case/control designs of human clinical/nutritional intervention studies should be complemented by longitudinal crossover studies, in which every subject is one’s own case and control. Human clinical study subjects should not only be assessed at homeostasis, but also during a challenge to, and restoration of, homeostasis.
(c) *Tools for molecular phenotyping and capturing of human diet and lifestyle:* nutrigenomic studies have typically been technology-driven rather than technology-rooted. Normative science methods and approaches need to be complemented by more comprehensive systems biology-based investigations deploying a multitude of omic platforms in an integrated fashion (41). While comprehensive and quantitative omics are rapidly progressing in terms of data generation, quantitative capture and monitoring of diet and lifestyle have lagged behind. Non-invasive technologies are now providing more attractive and precise image- and web-based or body-wearable consumer/research interfaces (42). The bottleneck in knowledge generation has moved from (omics and clinical) data acquisition to processing, visualization, and interpretation. Innovative tools and methods for statistical treatment and biological network analysis are now at the forefront of nutritional and biomedical sciences (43).

To achieve this transformation and advancement of nutritional science, it is critical to connect researchers from all disciplines conducting direct or indirect research in the areas, e.g., (gen)omics, clinicals, dietetics, food science and technology, physiology, epidemiology, bioengineering, analytics, biomathematics. A *transdisciplinary* approach needs to be considered – enabling a spectrum of communicating and sharing from fundamental laboratory research, patient- and consumer-relevant outputs from personalized dietary/nutritional counseling to monitoring/diagnostics. Progress in advancing nutrigenomic interventions for consumers and patients can only be accelerated if nutrition research is broadened to include quantitative, holistic, and molecular sciences (44).

“Let the food be your medicine, and medicine be your food,” a statement attributed to Hippocrates, the father of Western Medicine, delineates the impact of nutrition in human health and disease. Indeed, several decades of research at the interface of nutrition and immunology demonstrate that infectious, immune-mediated and metabolic diseases are safely and effectively preventable through dietary interventions. Nonetheless, there is a major disconnect between the description of nutrition-based protection from disease and an insufficient mechanistic understanding at the systems-level of the complex network interactions by which nutrition mediates clinical protection. As a result, a comprehensive understanding of the mechanisms of action underlying the actions of nutritional interventions and the combinatorial effects of nutrients (i.e., synergistic, antagonistic, or additive) at the systems-level remains largely unknown. As about 70% of the immune system is located in the gastrointestinal tract since the gut mucosa houses the largest repertoire of immune cells and commensal microbiota that symbiotically coexist to elicit protective immunity, studying nutritional immunology of the gut mucosa is incredibly important (45). Coupling host-nutrient-microbiota actions, enabled through computational modeling of the gastrointestinal tract (46–50) with systems immunology frameworks has the potential to predict combinatorial outcomes of nutrient-microbiota–immune system interactions and advance toward a comprehensive systems-level mechanistic understanding of how nutrition and foods prevent disease. Computational models of nutritional immunology that funnel omics and cellular data judiciously, coupled with systems biology models of the underlying disease/organ, will bridge the connection between traditional methods of nutritional immunology research and their effect on the whole organism, which will enhance mechanistic insights and translational value. Over 163 nutrition themed systems biology markup language models (SBML) are already available in the Biomodels database (51). In summary, applying the iterative systems biology cycle of model building, calibration, refinement, and validation in nutritional immunology research has the potential to accelerate the discovery of novel network biomarkers and systems-level mechanistic understanding of the action of dietary components on immune responses.

There has been an explosion in data collection and aggregation, some of which can be used for public health purposes, including obesity and nutrition-related research. Consequently, ample opportunities emerge to utilize “big data” in the pursuit of interesting outcomes and effectiveness studies related to nutrition and obesity using techniques such as quasi-experimental approaches. These approaches, when assumptions are satisfied, are intermediate between ordinary association tests and randomized controlled trials (37) in terms of presenting evidence for causality. In this article, the term “big data,” which is often used subjectively, refers to very large amounts of data: structured and unstructured that may also increase over time rapidly (52). These types of data are collected by both the public and private sectors and increasingly require a distributed architecture to manage them efficiently. Big data analysis has generally referred to the confluence of statistical, machine learning and computational approaches to synthesize and analyze these large amounts of data. Administrative data, such as micro-level data aggregated by governments as well as private companies, can be used to evaluate the effectiveness of pharmacological and surgical interventions. In fact, private companies have started collecting unprecedented amounts of data with some companies specializing in data linkages. For example, companies such as Optum not only aggregate claims data from private insurance companies but are able to provide linked clinical data from the corresponding electronic health records (EHR). Data linkages are an extremely powerful tool since they allow researchers to answer questions that are otherwise not accessible using a single data source. For example, claims data do not provide information about the height and weight of an individual, but the linked clinical data do. Similarly, the increasing availability of EHR data and the initiatives to link these EHR data with genomic data can enable us to pursue a variety of studies, including pharmacogenetic and precision medicine studies. One of the challenges in accessing and leveraging “big data” is the resources, including the associated cost of purchasing the data, especially from private companies. Collaborations between industry and academic researchers are essential to fully exploit the data and to overcome logistical challenges (53, 54).

So far, big data analysis has primarily focused on high-dimensional prediction models. The data mining and statistical toolkit for such approaches includes, but is not limited to, techniques such as boosting, random forests, classification and regression trees, and lasso-like penalized regression models (53). While randomized control trials are considered gold standards, there are a variety of methods and designs that may allow us to generate evidence that may lie in the spectrum between purely association and definitively causal. Coupled with “big data” is an opportunity to estimate a degree of causality using techniques such as high-dimensional propensity score and differential comparison approaches to provide evidence that is indicative of causality (55, 56). There is also a potential to use instrument variable approaches, used commonly in health policy studies, by identifying appropriate instruments from “big data.” Recent attempts to develop methods that enable to provide a degree of causal evidence are very encouraging and can allow us to maximize the potential of “big data” (57, 58).

## Authenticity and Safety of Foods

(Michael Rychlik)

The authenticity of food is generally related to one or more of the following attributes: geographic origin, type of agricultural production, species and kind of raw materials, or certain process qualities such as sustainability or ecological foot print.

Regularly uncovered crises of food adulteration underline the sensitivity of consumers to this issue. Apart from meat, foods that are often adulterated are olive oil, fish, organic foods, spices, tea, cocoa, coffee, and nuts.

In recent years, there has been tremendous progress in high-resolution methods to elucidate the molecular fingerprint of food. On the genetic scale, apart from classical polymerase chain reaction, new developments of isothermal amplifications or next generation sequencing will enable more accurate identification of species.

On the protein level, specific biomarker peptides can be used. For a fingerprint of metabolites, the new methods of non-targeted and targeted metabolomics already allow a specific authentication. In this field, the methods currently showing the best resolution are Fourier transform ion cyclotron mass spectrometry (FT/ICR-MS) or nuclear magnetic resonance (NMR) spectroscopy (59). These new methodologies generate “big data,” from which the relevant information is only accessible when applying novel bioinformatics approaches.

Regarding food safety, microbiological decay and foodborne infections still play an important role. However, contaminants also endanger the safety of all links in the whole food chain. The recent discoveries of process contaminants encompass simple molecules, such as acrylamide, furan, benzene, styrene, as well as more complex compounds such as 3-monochloropropane-1,2-diol (MCPD) esters. An end of new discoveries cannot be foreseen yet and we may assume that the sum of all these contaminants has a significant impact on life-style diseases such as cancer. Further new contaminants arise from packaging materials such as mineral oil saturated hydrocarbons (MOSH) or mineral oil aromatic hydrocarbons (MOAH), and pollutants from the environment such as the polyfluorinated alkyl substances (PFAS). Moreover, the historic toxin arsenic is more relevant than ever as rice and rice products are often contaminated and the mechanisms of arsenic carcinogenicity are still under controversial discussion.

Generally, risk assessment of food contaminants or residues is predominantly performed on single compounds. However, almost completely missing is an assessment of the combined effects of toxins, be it within one group of compounds or spanning various structural groups. The current concept for assessing combinatorial effects is that of cumulative assessment groups (CAGs), which, e.g., assess the cumulative potency corrected dose of acute reference doses (ARfD) for pesticides showing the same mode of toxic action (60). However, this approach is still preliminary and lacks comprehensive confirmation.

## The Science Behind Food-Related Behavior in Humans

(Adrian Meule, Chor San H. Khoo, and Claus Vögele)

Numerous environmental, social, and individual factors influence human food choice and intake (61). In Western and Westernized societies, household expenditures and dietary energy availability decreased for unprocessed or minimally processed foods in the last decades while they increased for convenience foods and processed products (62, 63). An environment where there is easy and frequent accessibility to food, and where cues signaling food are ubiquitous, requires constant self-monitoring and -regulation in order to prevent or manage weight gain (61). This, however, can be a highly effortful endeavor, leading many people to struggle with long-term weight maintenance. As evident from data from the last century, these self-regulatory efforts are made more difficult by increased consumption of energy-dense palatable foods and ingredients (e.g., sugar, fat, and salt) (64). As a result, some have argued that these foods might have an addictive potential and that a subset of individuals who have difficulties in controlling consumption of these foods may be addicted to them (65–68).

In the scientific literature, the association between food and addiction and the actual use of the term *food addiction* has a long history, dating back to the 1950s and even earlier times (69, 70). Not until recently, however, have researchers tried to more precisely define what is meant by food addiction and to systematically investigate its validity, as a consequence of which the number of publications, including the term food addiction, increased substantially over the past 5–6 years (65, 71). In humans, research on food addiction has been promoted by the Yale Food Addiction Scale (YFAS), a self-report questionnaire developed in 2009, which measures symptoms of addiction-like eating based on the diagnostic criteria for substance dependence as outlined in the fourth version of the Diagnostic and Statistical Manual of Mental Disorders (DSM-IV)(72). Since 2013, these diagnostic criteria have been revised in the fifth version of the DSM and a new version of the YFAS, which has been adapted accordingly, is currently under way (73).

Although research on food addiction is growing, it remains a controversial and debated topic with many researchers questioning the validity of the food addiction concept based on conceptual considerations or physiological mechanisms (74–78). To address these issues, more and better human studies are needed to resolve questions related to, for example, whether animal models of food addiction are transferable to human eating behavior (79, 80). These controversies, in particular, lead us to argue that food addiction research in humans is still in its infancy, that it would be premature to conclude that some foods are addictive, and that research efforts to clarify this will further increase in the years to come.

There are numerous avenues for future directions, which may include, but are not limited to: how do we define and harmonize definitions of food addiction? What are the implications of changes in the diagnostic criteria for substance dependence in the DSM-5 for food addiction (73)? Are all addiction criteria (as described in the DSM-5) equally applicable to human eating behavior? If not, does this obliterate the concept of food addiction (81)? How can food addiction be measured in humans other than using the YFAS and which methodological improvements need to be made to better design human behavior studies, including randomized controlled trials (72)? How relevant is the concept of food addiction for the treatment of obesity or binge eating and in public policy making? If it is relevant, how can it best be implemented (70, 82)? What are the disadvantages (if any) of the concept of food addiction (83–85)? How can animal models of addiction-like eating be improved to more specifically reflect relevant processes in humans (86)? Which foods are possibly addictive (87)? Can addiction-like eating actually be reduced to the addictive effects of substances or should the discussion about “food addiction” rather be replaced by a discussion on “eating addiction” (88)?

## The Molecular and Physiological Science Underlying Nutrition and Brain Health

(Pierre Magistretti, Johannes le Coutre, and Suzanne L. Dickson)

Cognitive decline, dementia, Alzheimer’s disease, and other age-related neurological diseases are on a rise in high income countries as well as in low and middle income countries (89). Achieving and maintaining brain health is a lifelong endeavor with identifiable targets that are specific for each period in a lifetime. Thus, targeting cognitive development in the early phases of life and preventing cognitive decline during aging are priorities for any preventive or interventional approach. While pharmacological approaches can only be envisioned for brief periods of time and, for the most part, have been unsuccessful, nutritional approaches are implementable for extended periods of time. Initiatives on brain health should incorporate a nutrition-based approach that can be implemented throughout the different phases of life.

In order to identify valid nutritional approaches for brain health, it is important to better understand the mechanisms that are at the basis of brain energy metabolism regulation. Key advances have been made in recent years in the identification of the molecular and cellular mechanisms that regulate the delivery of energy to active neurons. In particular, an active metabolic exchange has been characterized between neurons and astrocytes with specific molecular steps that can become targets for nutritional interventions.

For the identification of the efficacy of such nutritional interventions, means for appropriate monitoring of markers need to be defined. This can be achieved by monitoring with brain imaging techniques, structural markers with morphometric approaches and myelination with MR as well as functional activation with fMRI, PET, EEG, and MRS, coupled with neuropsychological tests monitoring cognitive performance, motivation, and attention. The utility of these technologies goes beyond brain health and many of these approaches are being used to validate, in humans, the neuroscience of nutrition that, so far, has only been conducted in rodent models (90, 91).

There is no doubt that targeting the molecular steps of brain metabolism with nutritional interventions and monitoring their structural and functional outcomes *in vivo* in humans, in particular regarding cognitive performance, represents a promising approach for developing nutritional interventions for achieving brain health that can be maintained on the long term. Meaningful nutrient intake and nutritional intervention likely has an impact on the development of cognitive and behavioral performance measures, thereby determining our health span throughout life. Brain imaging studies on infants demonstrate how breast milk promotes healthy neural growth and early white matter ­development (92).

Nutrients also engage brain pathways linked to metabolic control, appetite, and food-linked behaviors. There has been a general expectation that it must be possible to use food formulation/composition to control how much and what we eat by altering the satiating and/or reward value of food combinations (93, 94). Currently, we lack a sufficient scientific evidence base that certain “unhealthy” foods fall short of “healthy” foods in their ability to induce satiation, limit hunger, or reduce hedonic over-eating. Moreover, it has not yet been demonstrated that any food or combination of foods has beneficial effects on appetite and energy intake of sufficient duration or magnitude to impact on body weight or metabolic health (95). This is a new and emerging field for which major advances are likely to progress through a better understanding of how nutrients communicate with the appetite-regulatory brain networks. Nutrient-brain communication could be direct but likely engages intrinsic physiological control systems. For example, when we eat, sensing mechanisms in the gut signal information about the amount and content of the food to the brain by nervous and endocrine afferent signals. Indeed, gut-derived hormones such as ghrelin and glucagon-like peptide 1 communicate with hypothalamic and brainstem areas linked to energy balance but also to brain areas processing the reward value of food and even brain areas linked to emotion and cognition (96, 97). Thus, while it seems clear that appetite-regulating hormones have a capacity to redirect behaviors important for governing how much and what we eat, the extent to which nutrients can control these behaviors through engaging intrinsic endocrine signals remains to be elucidated.

A related question is whether specific nutrients or food combinations can act on the brain to reinforce their own intake, leading to addictive-like over-consumption. As reviewed recently (88) and as mentioned already in the previous section, it is very difficult to demonstrate in humans or rodents that foods act on the brain in a manner similar to addictive drugs, causing individuals to become addicted to them. It was suggested therefore that the term “eating addiction” rather than “food addiction” should be used to better describe addiction-like behavioral over-eating disorders. If it becomes possible to diagnose this patient group, e.g., through combining questionnaires about addictive-like behavior for food with brain imaging (98, 99), there will be a large public health impact on treatment and prevention strategies. Additionally, industrial stakeholders and politicians will need to find solutions to circumvent or treat eating addiction (88).

## The Science of the Human Microbiome

(Dietrich Knorr and Chor San H. Khoo)

The human body harbors over 8 million microbial genes, over 10,000 species, and plays host to over a trillion microbes. Microbial cells outnumber human cells by a factor of 10 (100). As a result, there is considerable interest to better define and understand the microbial role in host physiology, health, and disease etiology. In the last decade, there has been a tremendous surge in microbiome research funded by programs such as the Human Microbiome Project (HMP) and the MetHIT Program. Advancing new and multiple technological approaches – whole genome sequencing, metagenomics, high-throughput-analysis, proteomics, transcriptomics, cultivation, metabolomics, and bioinformatics – has led to new insights into microbial variety and abundance in 15–18 body sites, including the oral cavity, skin, airway, gut, and vagina, from 242 healthy participants in the largest cohort study to date. Findings from this research were published in two seminal papers in 2012 by the Human Microbiome Consortium (100, 101). The HMP study has the largest collection of data on abundance and variety of the human microbiome, with 5,177 unique microbial taxonomic profiles from 16S ribosomal RNA genes, more than 3.5 terabases of metagenomic sequence, and 800 reference strains isolated and sequenced (100). Noteworthy observations from the HMP study are outlined in Table 2 (102).

**Table 2:** **Variation in microbial ecology among individuals (102)**.

Each person’s microbiome is unique and no two individuals have the same microbiome (102). However, in spite of individual microbial differences, different individuals can still be considered healthy
Microbial communities across varying body regions may predict some characteristics such as breast fed history and educational level
Microbial communities from different body regions from an individual were predictive for others. For example, the oral community can be used to predict the gut community
Overall, low relative numbers of pathogens have been observed
Strong site specialization but considerable variation in diversity and abundance of each habitat’s signature microbes among subjects
Strong functional stability. This means that while the microbial compositions were widely different, the functionality is similar. This suggests flexibility to develop microbial communities that can provide similar performance
Wide variation in patterns of alpha and beta diversity (alpha-diversity within a site; beta diversity among subjects)
Correlations between ethnicity and microbiome composition across all body habitats
A positive correlation of vaginal pH to microbial diversity (higher pH having higher diversity)
An association of age with skin microbiome-associated metabolic pathways and oral microbiome composition

Translating learnings from emerging microbiome and health research presents exciting opportunities for future food and nutrition development. The use of microbes in food product development is not new. Fermented products are widespread and common in the market place. Food biotechnology has been in existence for more than 8,000 years (103). The potential health impact of gut microbiota has been postulated by Metchnikoff (104) and since then, numerous related research results have been provided (105–107). Probiotics are supplied in starter cultures and thus need to be preserved for transportation and use. As the highest possible cell density is required, losses that occur during processing, transportation, and storage, including in products, are detrimental. Consequently, approaches to increase and retain physiological fitness have been explored (108, 109).

Emerging capabilities to characterize microbial communities and their functions in the oral cavity present insights into the role microbes may play in taste and olfaction, and present new opportunities to further personalize and refine food products to better suit individual taste and palatability preferences. Oral pre- and probiotics may be an opportunity for innovation.

These emerging advances in human microbiome structure, diversity, and function present exciting new opportunities for new food products, ingredients, or dietary approaches that can be used for supporting daily health, direct or adjunct intervention for risk reduction, or for new therapeutics for symptom reliefs (IBS). However, to advance these undertakings, several key questions need to be addressed. How easy is it to translate microbiome research to food and dietary applications? Limited well-designed studies have been performed that explore the impact of food and diet on microbial ecology and function. What biomarkers are available or need to be developed to understand how food and diet impact on the microbiome (gut, gut-brain, gut-kidney, etc.)? What microbial combination will be best suited for achieving specific outcomes? Of challenge is the ability to identify and separate the “good” from the “bad” microbes that can present food borne illness or exacerbate disease risks. Gene sequencing and whole genome sequencing technologies have been used to diagnose and trace food contamination, and are now also applied in medicine. How can current microbiome research be easily translated for food and product applications? How easy is it to transfer available technologies and tools already developed for use in food and nutrition applications?

In addition, there remains room for improvement when translating to innovative or tailor-made products. Needs and opportunities include process generated structures, which impact on food properties (process–structure–function relationship) as outlined in the European Technology Platform Strategic Research Agenda (ETP SRA) (2007; 2012; 2014) for designing tailor-made foods for the targeted release of essential food constituents at points of need to support human microbiota growth and metabolic fitness. This needs to include the entire human digestion system encompassing the chewing apparatus, mouth microbiota, and enzymes. Moreover, food can contain viable microbial cultures as well as active enzymes. Understanding their role in and during digestion as well as their impact on gut, mouth, and skin microbiota may lead to the development of new food design concepts with targeted nutritional benefits.

Finally, emerging technologies are being introduced to the food processing area, including high hydrostatic pressure, pulsed electric fields, and atmospheric plasma. Little is known about their impact and function with regard to the human microbiota. These technologies could open new avenues for process–­function–structure relationships as well as provide foods with metabolic properties not achieved via traditional processing (36).

## Nourishing the Immune System and Preventing Disease

(Johan Garssen, Willem van Eden, and Josep Bassaganya-Riera)

Whereas the disciplines of pharmaceutical and nutritional sciences have evolved separately in the Western world, for Asia these two research areas have been connected for centuries. However, today, with the ever-growing burden of chronic diseases in modern societies, the high relevance of specialized nutrition in both prevention and therapeutic approaches receives increased attention and recognition. The gap between food and pharma is narrowing (110). One reason might be that, scientifically, the evidence for the so-called multi-target or polypharmacology approaches aimed at disease management is growing. Medical nutrition is beginning to be recognized as a unique and potentially powerful area in Western societies at the interface between food and pharma.

Medical nutrition targets innovative nutritional therapies, offering healthcare professionals solutions to effectively manage disease-related malnutrition and specific disease states. Medical nutrition is and will be increasingly understood as a useful and sometimes even essential component in the management of patient health. Many medical conditions can be managed better when patients are receiving a specialized diet adapted to their unique circumstances. Sometimes, the constraints to appetite may be physical, as in the case of stroke patients who may find it difficult or impossible to swallow, or of young children with neurological disabilities. Sometimes, the problem may simply be insufficient intake, caused by the loss of appetite. It is well known that many chronic diseases are associated with malnutrition, a phenomenon that is not solely based on body mass index or body weight. Many obese patients suffer from specific malnutrition. Examples of disease areas that might be associated with specific malnutrition are cancer, stroke, and COPD. However, frail or elderly people are treated and fed with this type of medical nutrition as well. Medical nutrition might bring solutions and support to these cases across a broad range of care settings – in the hospital, in the care home, or in the community. It contains unique compositions of specific nutrients that would be impossible or impractical to achieve through normal food intake alone. In most cases, it is administered via the gastrointestinal tract orally or with a feeding tube, utilizing the natural route for nutrient digestion and absorption. These cases are underpinned by a unique scientific rationale, preclinical and clinical research, and health economic evaluation making it very similar to the traditional pharma approach. By making medical nutrition an integral part of care, patient outcomes are significantly improved. Lower healthcare costs by shortening hospital stays and keeping patients independent for longer are key outcomes for medical nutrition intervention. The food for special medical purposes (FSMP) is the regulatory directive involved with the quality/safety and efficacy of medical foods.

Another and unique medical area for which medical nutrition is aimed is disease-specific (the so-called disease targeted) medical nutrition. This type of medical nutrition is a unique, effective, therapeutic nutritional intervention for patients with, e.g., a clinical need to avoid certain nutrients due to specific diseases or conditions where normal food intake is harmful. Examples are inborn errors of metabolism such as phenylketonuria (PKU) or severe cow’s milk allergy and childhood epilepsy. Ketogenic therapy during refractory epilepsy can reduce seizures significantly. Other examples for disease-specific medical nutrition are science-driven concepts containing different and uniquely selected nutrients that can act in an orchestra leading to a delay in disease progression. Validated examples have been described for Alzheimer’s, HIV, diabetes, and cancer (111–114).

Disease-targeted medical nutrition can be aimed at conditions such as chronic inflammation. These inflammatory conditions are on the rise. This is caused by changes in life-style, food consumption patterns, and aging. Inflammation-associated conditions, such as atherosclerosis, type 1 and type 2 diabetes, obesity, Alzheimer’s disease, and many others, are a growing burden to health budgets. Inflammatory conditions are thought to result from failing mechanisms of immunological tolerance. Of these mechanisms, deficient suppressive activities of a specialized subset of T cells, called regulatory T cells (Tregs), are being recognized as a major factor in the failure of immunological tolerance. A start has been made with the definition of antigen-specific Tregs with a broad anti-inflammatory effect, such as, for example, those that recognize inflammation-associated stress-proteins (115). Herewith, the restoration of this regulation will be a widely sought goal, also for the field of nutrition. A telling example of what may be possible is the following. Wieten et al. have shown that the up-regulation of stress-proteins, such as heat shock protein 70 (HSP70), in the cells lining the gut, leads to the local induction of Tregs (116). Working with a model of chronic and relapsing arthritis, it was found that HSP70 was also induced in Peyer’s patches and the induced HSP70-specific Tregs were having a systemic effect seen to fully control arthritis. This up-regulation was achieved by the oral administration in mice of carvacrol, an essential oil of Oregano species. It showed that our diet may contain effective co-inducers of stress-proteins and that these co-induced proteins can elicit anti-inflammatory activity in the immune system. Similar activities have now been described for other food components (117). Therefore, especially for the diets of the aging individual, substances with anti-inflammatory activities will be an attractive component. In the field of veterinary medicine and food animal production, restrictions are now being imposed on the use of antibiotics, certainly on the use of antibiotics as growth-enhancers. Also here, feed additives are searched with the purpose of controlling inflammation and thereby enhancing weight gain.

In combination with drugs, medical devices and lifestyle modification, medical nutrition, and immune system targeted nutraceuticals can play an essential role in health care and precision medicine. Expectedly, it will lead to lower costs of care: fewer complications, shorter hospital stays and reduced mortality, and the reduction of disease manifestations.

Over the coming years, Medical Nutrition and Nutraceuticals have the opportunity to be accepted as a bridge between food and traditional pharma approaches – not as isolated therapy but as part of integrated systems-wide health care. Additionally, pharma often is focusing on a monotherapeutic approach (one molecule one target) and medical nutrition will be recognized as the multi-target approach for disease management. Regulation and acceptance depends on national and international guidelines. Changes in regulation for medical nutrition are to be expected since medical nutrition is a relatively new therapeutic area that falls between different regulations and guidelines. For instance, in the USA, under section 5(b) of the Orphan Drug Act [21 U. S. C. 360ee (b)(3)], a medical food is formulated to be consumed or administered enterally under the supervision of a physician and which is intended for the specific dietary management of a disease or condition for which distinctive nutritional requirements, based on recognized scientific principles, are established by medical evaluation. Thus, from a regulatory perspective, medical foods are different than dietary supplements in that claims for medical foods can allude to disease management whereas dietary supplement claims cannot. Medical foods are exempted from the labeling requirements for health claims and nutrient content claims under the Nutrition Labeling and Education Act of 1990. In order to be a medical food, a product must meet the following criteria: to be a food for oral or tube feeding, the product must be labeled for the dietary management of a specific medical disorder, disease, or condition for which there are distinctive nutritional requirements, and the product must be intended to be used under medical supervision. Essentially, medical food comes into play when dietary management cannot be achieved by the modification of the normal diet alone. For instance, medical foods could be used to replete key metabolic components that might be depleted in diabetes or inflammation. Only translational research and randomized, placebo controlled double-blind clinical trials can validate these new concepts.

## Conflict of Interest Statement

The authors declare that the research was conducted in the absence of any commercial or financial relationships that could be construed as a potential conflict of interest.
